# A Newly Developed Easily Sinterable Low-Alloy Steel Powder

**DOI:** 10.3390/ma14020406

**Published:** 2021-01-15

**Authors:** Dorota Tyrala, Janusz Konstanty, Izabela Kalemba-Rec

**Affiliations:** Faculty of Metals Engineering and Industrial Computer Science, AGH University of Science and Technology, 30 Mickiewicz Avenue, 30-059 Krakow, Poland; konstant@agh.edu.pl (J.K.); kalemba@agh.edu.pl (I.K.-R.)

**Keywords:** sintered diamond tools, good sinterability, low-alloy steel powder

## Abstract

The work presents a possibility of fabrication of inexpensive iron-based powders intended to form the matrix in sintered diamond-impregnated tool components. In this study, a finely dispersed, pre-alloyed steel powder, containing over 95 wt.% Fe, has been designed and fabricated by means of a proprietary process developed at AGH-University of Science & Technology. It has been shown that the experimental powder can be consolidated to a closed porosity condition (>95% theoretical density) by pressure-less sintering at a temperature below 900 °C. The as-consolidated material is characterized by an excellent combination of hardness (~250 HV) and mechanical strength (>1200 MPa in 3-point bending) that meets the diamond tooling requirements. Its properties can be modified to some extent by varying the cold forming pressure and sintering temperature.

## 1. Introduction

Low-alloy steel powders have been found useful in the manufacture of numerous high performance sintered parts. Unlike structural materials, where sintering at 1120 °C is acceptable and closed porosity is rarely a critical concern, near-full densification of diamond tool components at a markedly lower temperature is crucial for their performance. In the past, the hot pressing technology was primarily used in the diamond tool industry due to the ease of reaching virtually pore-free condition. Disadvantages of pressure assisted techniques are high cost and limited productivity. Therefore, the conventional cold press/sinter route is rapidly gaining in popularity, especially in the fabrication of wire saw beads [1,2,3,4,5].

A serious shortcoming of pressure-less sintering of high-density parts is the prolonged exposure to high temperature, which may have harmful effects on diamond crystals [6]. Hence, much work has recently been done [5,7,8,9] to develop new matrix powders, which could be sintered to a high density at maximum 900 °C. The material thus produced should also meet some other application criteria, such as high hardness, mechanical strength, toughness, and resistance to abrasion [10].

To date, two iron-base powders have been offered commercially and achieved large-scale industrial use [11]. Their nominal chemical compositions and rough indication of properties are given in Table 1. 

Both these powders satisfy the 95% theoretical density (TD) criterion after sintering at 900 °C, but other fine powders (e.g., Fe-P) have to be admixed to increase the as-sintered hardness to minimum 92 HRB (>200 HV) [14,15,16]. Therefore, the objective of the present work was to design and manufacture a low-alloy steel powder, which would combine excellent sinterability with high as-sintered hardness and strength.

The recent powder developmental efforts have been aimed at designing an alloy that would show a fine-grained, multi-phase structure at around 900 °C [7,8,9,10]. Copper, nickel, and phosphorus have been selected as alloying elements because of their easily reducible oxides. Copper stabilizes austenite and has limited solubility in iron, which enables formation of a separate (Cu) phase. Nickel is also an austenite stabilizer. It moderately contributes to solid–solution strengthening of both the (Cu) phase and ferrite, and improves ductility of ferrite. Contrary to copper and nickel, phosphorus is a ferrite stabilizing element, which produces a narrow γ loop in the Fe-P phase diagram. Among all alloying elements, phosphorus is the most effective solution strengthener in steels [17,18]. 

It is well established that a combination of fine-grained structure and placement of fine pores on grain boundaries, or interphase boundaries, aids in pore shrinkage [19,20]. Under such conditions, pores “dissolve” by diffusing vacancies to adjacent boundaries. Densification proceeds rapidly until separation of boundaries from pores occurs. Therefore, the sintering cycle requires precise planning and execution in order to ensure high boundary diffusion rates and to avoid grain growth leading to pore isolation. It seems likely that a combination of relatively high green density and narrow pore size distribution within the green body, as well as fine particle size of a polycrystalline, multiphase powder should lead to 95–98% of the pore-free density after a ½ hour hold at ~900 °C in hydrogen. Further densification seems impractical because longer sintering cycles, needed for removal of gas entrapped in isolated pores, shall lower productivity.

## 2. Materials and Methods

Using the ThermoCalc software (version 2014), numerical simulations were repeatedly carried out until the amount of alloying elements was reduced to 4 wt.% and a well balanced multiphase structure was achieved within a relatively wide sintering window located below 900 °C. A detailed analysis of time–temperature profiles in a typical three-zone conveyor belt furnace showed that the peak temperature control to within ±20 °C was possible when the belt speed and hot zone temperature were preset to realize ½ hour hold at ~900 °C [21]. Therefore, assuming that a scatter of ±10 vol.% on either phase does not compromise the ability to resist grain growth, the sintering window was defined as a 40 °C wide peak temperature range in which volume fractions of both ferrite and austenite were kept between 40 and 60%.

To validate the above theoretical assumptions, it was necessary to perform experiments on sintered compacts. To this end, an experimental steel powder containing 2.3% Cu, 1% Ni and 0.7% P was manufactured using a proprietary process developed at AGH-UST [9]. Prior to consolidation, the powder was tested for particle size and shape, specific surface area, apparent and tap densities, hydrogen loss, and phase composition. Measurements of apparent density by the Scott volumeter method, tap density, specific surface area, and loss of mass on hydrogen reduction were carried out in compliance with relevant ISO standards [22,23,24,25]. The Subsieve Auto Sizer of Particulate Systems (Norcross, GA, USA) used for determination of specific surface area was basing on the Kozeny–Carman formula and allowed rapid calculations of both mass-specific surface area and mean particle size. The particle size distribution was estimated using the Winner2000B laser particle size analyser (Jinan Particle Instrument Co., Ltd., Jinan, China), and the Mie theory of light scattering, in compliance with reference [26]. The data were generated by the Winner2000B software package and displayed on a volume basis.

Knowing the Scott density, an appropriate amount of powder was volumetrically fed into a 12 mm × 40 mm cavity of a carbide-lined die in order to obtain green samples ranging from 4 to 5 mm in height after cold compaction at between 200 and 600 MPa. Neither die wall lubrication nor dry lubricant addition was used. Prior to sintering, all green compacts were measured with a digital micrometer and weighted to determine green density. 

Sintering was performed in a laboratory tube furnace in a hydrogen atmosphere. Two green compacts were placed side by side on a ceramic plate and sintered together for 30 min. A temperature-monitoring thermocouple was positioned adjacent to the compacts. During heating, the green parts were held for 30 min. at 700 °C, for oxide reduction, before proceeding to the sintering temperature. 

After cooling to room temperature, the sintered parts were tested for density, bending strength, hardness, and phase composition as well as subjected to metallographic observations on both fracture surfaces and metallographic cross sections. 

The as-sintered densities were measured, basing on the Archimedes’ principle. 

**Figure 1:**
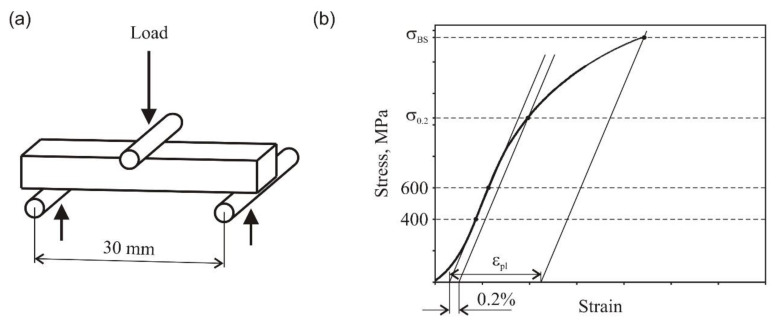
Three-point bend test fixture (**a**) and evaluation of bending strength (σ_BS_), offset yield strength (σ_0.2_), and plastic strain at failure (ε_pl_) from a bending curve (**b**) [27].

The bending strength was tested with nonstandard conditions. The sintered 3 mm × 10 mm × 40 mm bend samples were supported by two high speed steel (HSS) rods, 3.5 mm in diameter, and lying 30 mm apart. The load was applied using a similar HSS rod positioned midway between the supports. The traverse speed was 0.5 mm/min. The three-point bend test setup and experimental procedure used for calculation of material property parameters are explained schematically in Figure 1.

Hardness was determined on metallographic sections by means of the Innovatest Nexus 400 Micro-Vickers/Knoop tester (Innovatest Europe BV, Maastricht, The Netherlands) using the Vickers hardness scale at a 1 kgf load. 

X-ray diffraction (XRD) experiments were carried out in order to determine phases and assess the volume fractions of copper-base solid solution (V_(Cu)_) and lattice spacings (a_fe_) of ferrite both in the powder and in samples sintered at various temperatures. The XRD data were collected on the Model D500 Siemens/Bruker diffractometer (Bruker AXS GmbH, Karlsruhe, Germany) using CoKα radiation in a step-scan mode with a step 0.04° of 2θ and counting time 10 s.

The FEI Inspect S250 scanning electron microscope (SEM, ThermoFisher Scientific, Hillsboro, OR, USA), fitted with an energy dispersive spectrometer (EDS), as well as the FEI Nova NanoSEM electron backscatter diffraction (EBSD) system were used to execute all microscopic analyses. EBSD patterns were acquired at 15 kV with a step of 0.2 μm and subsequently processed using the OIM Analysis™ v7.1.0 software.

## 3. Results

### 3.1. Powder Design and Fabrication

Numerical simulations were repeatedly carried out until the alloy met all chemical composition and phase structure conditions described in Section 2.

**Figure 2:**
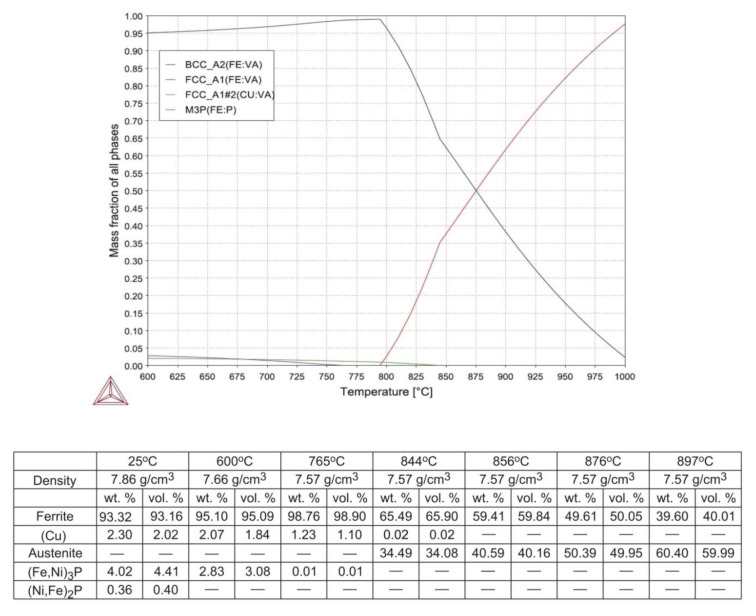
Effect of temperature on phase composition of the newly designed Fe-2.3% Cu-1% Ni-0.7% P alloy.

The final outcome of calculations is shown in Figure 2.

As seen in Figure 2, complete dissolution of (Fe,Ni)_3_P and (Cu) in iron takes place at 766 and 845 °C, respectively. The α^®^γ transformation begins at 795 °C and the amount of austenite increases steadily with temperature to reach around 62 wt.% (61.4 vol.%) at 900 °C. In order to most effectively inhibit grain growth during sintering, the sintering window should preferably be centred around 876 °C, where the austenite-to-ferrite volume ratio approaches unity. Assuming that a scatter of ±10 vol.% on either phase does not compromise the ability to resist grain growth, the sintering window becomes 41 °C wide, ranging from 856 to 897 °C.

### 3.2. Powder Characteristic 

The bulk properties of the experimental powder were determined according to the current ISO standards. The volume mean diameter (D[4,3]) was reported along with D10, D50, D90, and D99 values (e.g., D10: 90% above, 10% below D10).

**Figure 3:**
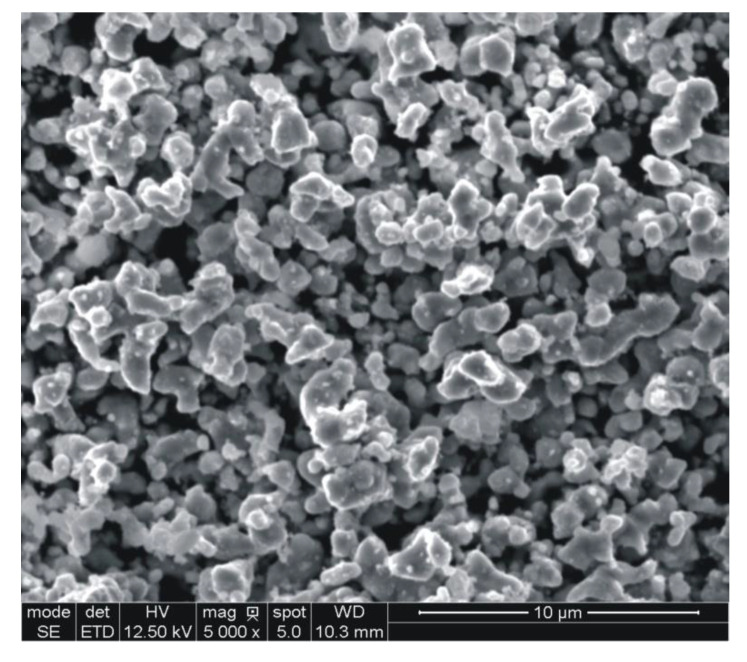
SEM micrograph of the experimental steel powder.

The results are presented in Table 2, whereas the powder particle morphology is shown in Figure 3.

### 3.3. As-Sintered Densities

All sintered parts were subjected to density measurements using the Archimedes method. Table 3 compares the obtained values with green densities assessed from the weight and linear dimensions of green compacts.

It is worthy of notice that all weight readings stabilized as soon as the sintered samples were immersed in water, indicating the lack of open porosity. Moreover, all tested specimens were blotted to remove surface water and reweighed in air after weighing in water. Again, no weight gain was observed, confirming that no water had entered the pores.

### 3.4. Mechanical Strength and Fractography

The sintered samples, which had been previously compacted at 400 MPa, were subjected to the proprietary three-point bend test. The results of measurements are presented in Table 4.

**Figure 4:**
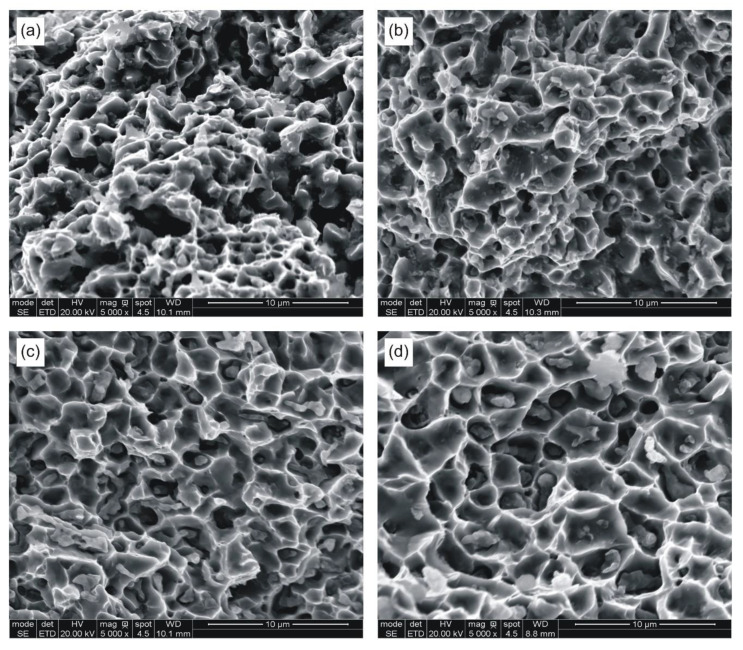
SEM fractographs of samples sintered at: (**a**) 850 °C; (**b**) 874 °C; (**c**) 898 °C; and (**d**) 924 °C.

The broken beams were examined microscopically prior to preparing metallographic sections. Selected fracture surfaces are presented in Figure 4.

### 3.5. X-ray Diffraction

Both powder and solid samples were used for quantitative phase analysis by XRD. The sintered samples were prepared by cutting out the central part of a bending bar using an alumina cut-off wheel and mounting it in Bakelite. The resulting sections were then wet ground on #220 SiC abrasive paper and successively polished on cloths impregnated with 9-, 3-, and 1-μm diamond compound.

**Figure 5:**
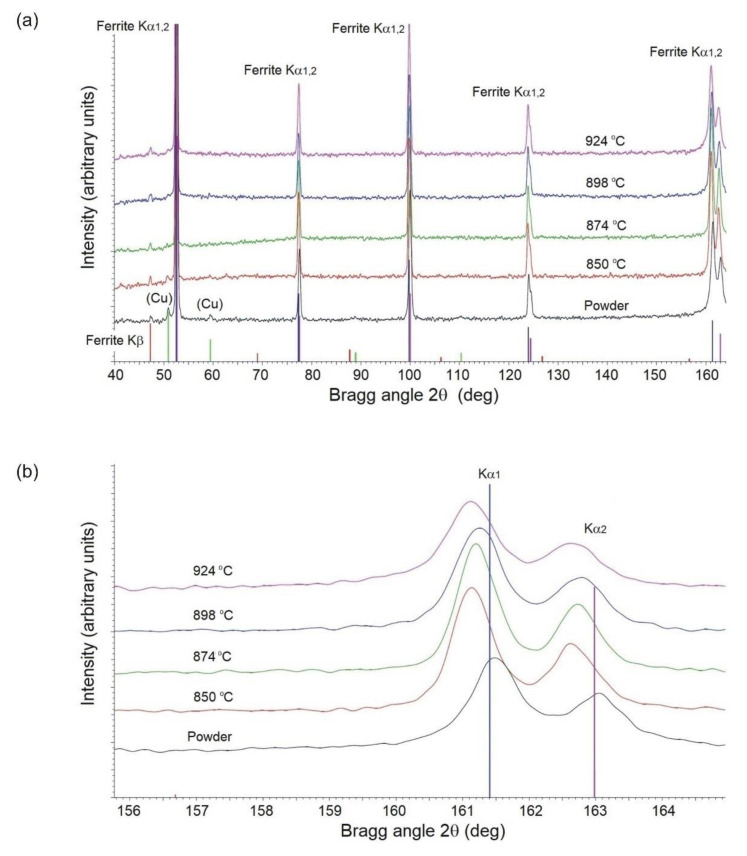
XRD patterns of the experimental steel (**a**) and a magnified view of the same graph showing positions of ferrite (310) peaks (**b**) used to calculate lattice parameters.

The results are presented in Figure 5 and in Table 5.

### 3.6. Vickers Hardness

After the XRD analysis the polished sections were used to measure Vickers hardness. The average values of ten readings are reported in Table 6.

### 3.7. Metallographic Examinations

After the hardness test, all metallographic sections were re-polished and mildly etched on the Struers OP-Chem cloth (Struers A/S, Ballerup, Denmark) with an active oxide suspension. Careful metallographic preparation was very important for further quantification of sintered porosity and grain structure.

**Figure 6:**
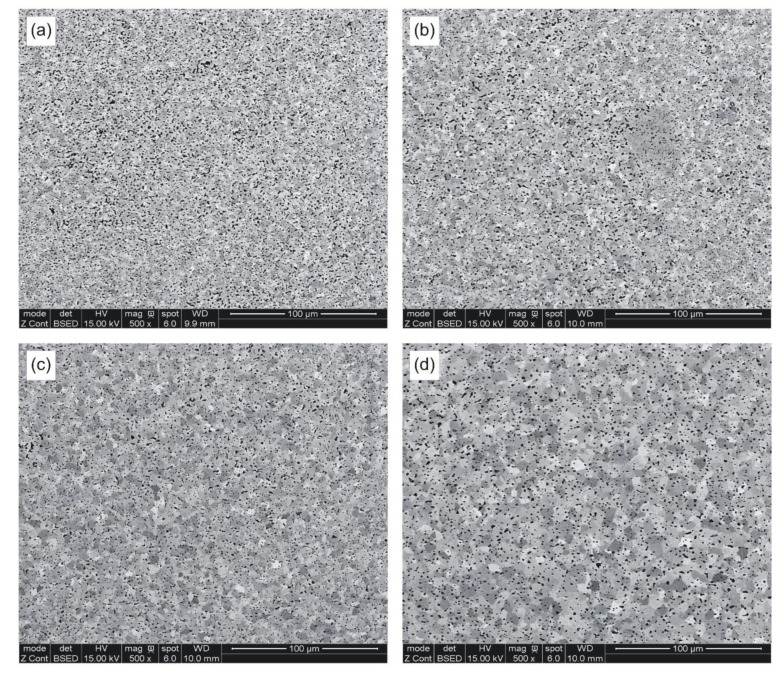
SEM micrographs of samples sintered at: (**a**) 850 °C; (**b**) 874 °C; (**c**) 898 °C; and (**d**) 924 °C.

**Figure 7:**
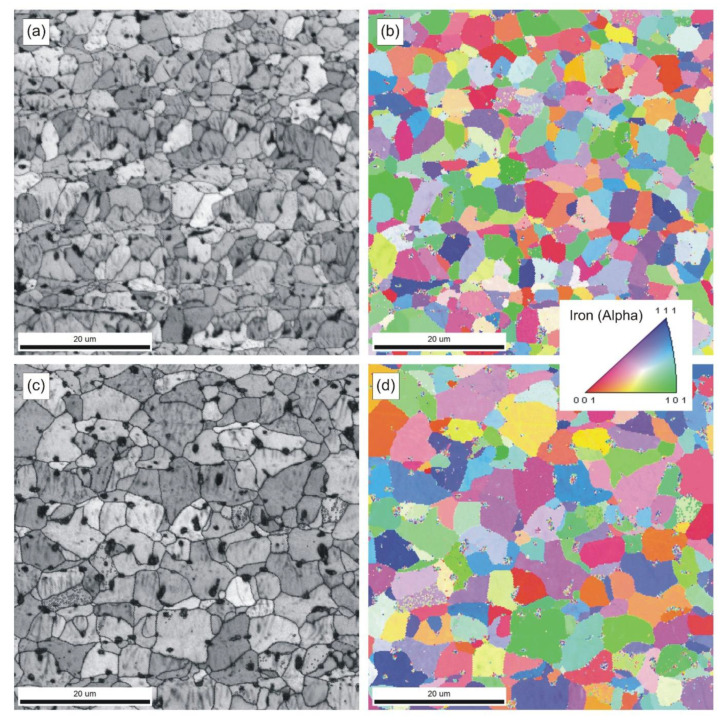
SEM EBSD micrographs (**a**,**c**) and inverse pole figure (IPF) maps (**b**,**d**) of samples sintered at: 898 °C (**a**,**b**) and 924 °C (**c**,**d**).

The resulting microstructures are shown in Figure 6 and Figure 7.

Figure 6 presents selected micrographs characteristic of samples sintered at various temperatures. Eight SEM micrographs were taken on each metallographic section at a relatively low 500× magnification, in order to obtain reasonably accurate quantitative information on the total porosity, pore morphology, average pore size, and size distribution. The standard ToupView camera image processing software was used to analyze the individual fields and store the data for subsequent calculations of planar pore size, number of pores per unit of test area (*P_A_*), and pore circularity. The latter parameter was obtained by dividing the pore area by its perimeter and multiplying by 4π to normalize its value to 1 for circles of the same size.

Figure 7 shows typical microstructures and grain orientation maps of samples sintered at 898 and 924 °C. The data collected with an electron backscatter diffraction (EBSD) system were also used to determine the grain size distribution. In this particular case, the analytical software calculated a project area diameter (*d_a_*) for each individual grain, i.e., a diameter of a circle with the same area as the two-dimensional image of the grain.

**Figure 8:**
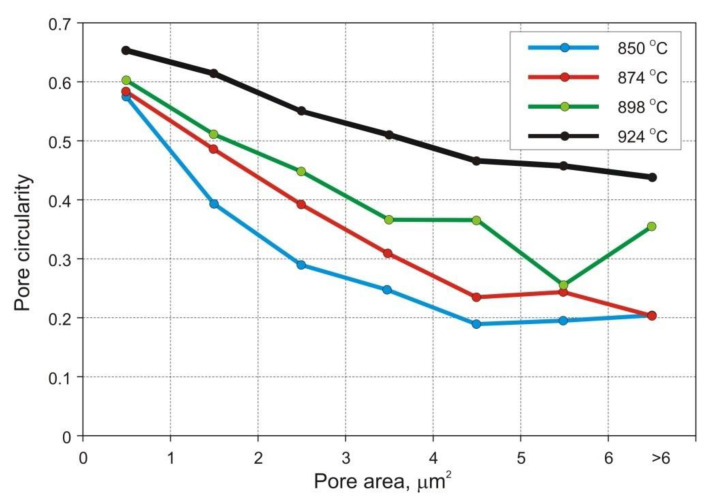
Pore circularity as a function of pore area.

**Figure 9:**
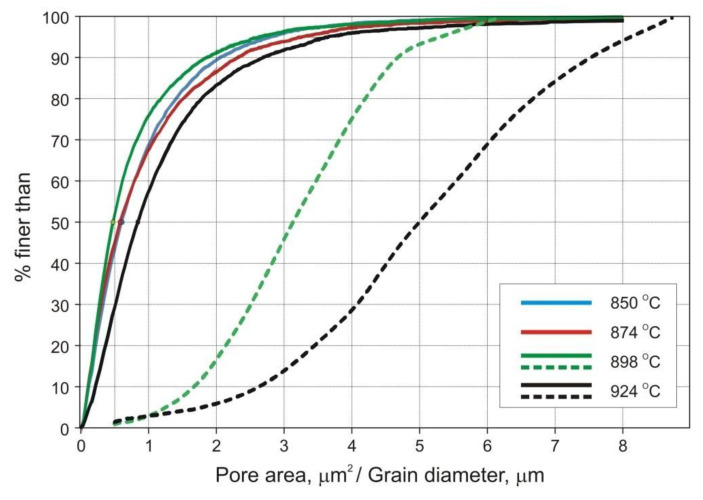
Cumulative plot of pore area (solid curves) and grain diameter *d_a_* (dashed curves) distributions.

The results are presented in Table 7 and in Figure 8 and Figure 9.

The EDS analysis was also performed by quantifying elemental composition of cross section areas seen at a magnification of 500×. Because the copper-base solid solution can be seen as a separate phase, the wt.% Cu was also converted to vol.% Cu.

The results are presented in Table 8.

## 4. Discussion

The results obtained in this study indicate that the experimental powder has been properly designed and manufactured to meet the application criteria that are favorable for fabrication of diamond tool components. As shown in Table 2 and Figure 3, the very fine, loosely agglomerated, low-alloy steel powder is characterized by a narrow distribution of particle size. The powder is fully pre-alloyed and has predominantly ferritic structure. The XRD and EDS data, given in Figure 5 and in Table 5 and Table 8, imply that the actual content of copper in the experimental powder exceeds the nominal value by around 1 wt.%. It means that complete dissolution of copper in ferrite takes place at around 875 °C, and not above 844 °C as suggested in Figure 2. Thus, higher concentrations of (Cu) in the powder and in samples sintered at 850 °C can be justified, whereas the minor differences between samples sintered between 874 and 924 °C are due to small sample-to-sample variation in chemical composition and cooling rate. As opposed to thermodynamic stability calculations presented in Figure 2, (Fe,Ni)_3_P has not been detected in the material as a separate phase and the whole of the phosphorus remains in solution in the ferrite. The atomic radii of BCC iron, phosphorus, nickel, and copper are 1.241, 1.1, 1.243, and 1.278 Å, respectively [28]. Therefore, the lattice parameter of ferrite rises with increasing copper concentration from 2.86582 Å in the powder to 2.86687–2.86737 Å in the sintered parts. This has been illustrated in Figure 5, by comparing positions of the experimental (310) peaks relative to the BCC iron standard. Phosphorus dissolved in the powder, having the smallest atomic radius, shifts both ferrite Kα1 and Kα2 diffraction lines to higher angles, whereas copper has the opposite effect when increasingly dissolved in ferrite after sintering. Due to the difference in atomic size between the iron and solute atoms (phosphorus, copper, and nickel) the alloying elements effectively contribute to solid solution strengthening of the sintered material.

It has been found that green parts, cold pressed to around 65% TD, can be pressure-less sintered to minimum 96% TD by a ½ hour hold above 870 °C in hydrogen atmosphere. The highest as-sintered density has been obtained after cold pressing at 600 MPa, however, compaction of diamond containing mixtures may prove impractical because of severe tooling wear. The as-sintered densities of samples compacted at 400 MPa range from 94.4 to 97.0% TD when measured by the Archimedes method. The respective values are even higher when determined metallographically; however, microscopic methods are sensitive to the sample homogeneity and preparation. Therefore, densities obtained from the Archimedes method are more reliable. Notwithstanding the density measurement technique, cold compaction of a granulated powder at 400 MPa seems to be best suited for fabrication of diamond tool components.

It is clear from Table 4 and Table 6 that the optimum sintering temperature lies between 874 and 924 °C. The samples sintered at 850 °C fail in a brittle manner showing large scatter in bending strength. As seen in Figure 4, the fracture surfaces show varying degrees of ductile failure with sintering temperature. The samples sintered at 850 °C partly fail along interparticle boundaries, whereas those sintered at 924 °C show ductile dimple morphology.

Improved ductility obviously has a bearing on strength and hardness of the material which increase considerably with increasing sintering temperature, due to structural changes that take place during sintering. It is noteworthy that the samples sintered between 874 and 924 °C are characterized by higher yield strength than hot pressed extrafine cobalt powder (σ_0.2_ = 1238 MPa [29]), although cobalt shows markedly higher bending strength (σ_BS_) and ductility (ε_pl_) [29]. Both of these properties can be markedly improved by sintering at 924 °C. The improvement comes at the expense of only slightly reduced yield strength and hardness.

The strength and ductility of sintered parts are explainable in terms of microstructural features, such as pore microstructure and grain size. The microstructures presented in Figure 6 and Figure 7, as well as data included in Table 7 and in Figure 8 and Figure 9 clearly demonstrate evolution of these two parameters with sintering temperature. It is evident from Table 7, that the temperature rise from 850 to 898 °C results in merely slight decrease in mean pore area accompanied by more evident decrease in the number of pores per unit area (*P_A_*) and increase in pore circularity. Apparently, the mean pore size decreases together with porosity until a certain degree of densification has been attained. At temperatures above 900 °C large pores begin to grow at the expense of smaller pores via Ostwald ripening. This is accompanied by pore rounding, as seen in Figure 8. Round pores provide improved resistance to crack propagation, thereby aiding ductility.

Pronounced microstructural changes are observed after sintering at 924 °C. The decreasing number of pores per unit area (*P_A_*) and markedly increasing pore size combine to lower effectiveness of grain boundary pinning by the pores. Thus, grain growth is promoted. These parameters have been quantified using image analysis techniques and are presented in Figure 7 and Figure 9. Despite increased pore size and pore spacing, little separation of pores from grain boundaries has taken place to form isolated pores, as seen in Figure 7c. Phase and grain boundaries are perfect vacancy sinks [20]. Therefore, even though the grains slightly grow at 924 °C, the sintered microstructure remains fine and densification proceeds, although at a markedly slower rate.

## 5. Conclusions

The results of the current study show that the newly developed and manufactured low-alloy steel powder can be effectively used for production of high-density parts by the conventional cold press/sinter route. Most commercial low-alloy steel powders require sintering at 1120 °C, or higher, to reach the closed porosity level. The experimental powder can be sintered to near-full density at merely 850 °C, although the best combination of hardness, strength, and ductility is reached after sintering at temperatures slightly higher than 900 °C. Because the density improvement between 874 and 924 °C is negligible, further gains in density and ductility by sintering above this range may not compensate for detrimental effects on both hardness and yield strength.

Interestingly, the new steel outperforms hot pressed cobalt in yield strength. This property is commonly considered as a primary factor that determines strong retention of diamond crystals in the matrix [30]. Taking into account the technical considerations included in this article, it is reasonable to conclude that economic benefits may also be achieved if this technique is brought to production scale.

## Figures and Tables

**Table 1:** Main properties of two powders designed for production of diamond tools by pressure-less sintering [7,12,13].

Powder Designation	Chemical Composition, wt.%						Fisher Mean Particle Size, µm	Density ^1^		HRB ^1^
	Cu	Co	Sn	W	Y_2_O_3_	Fe		Theoretical, g/cm^3^	Relative, %	
Next 400	35	15	-	-	-	bal.	2.0	8.35	98.8	89
Cobalite CNF	26	<0.5	3	2	0.6	bal.	2.0	8.18	99.0	85

^1^ after sintering for 1 h at 900 °C in 75% H_2_/25% N_2_ atmosphere.

**Table 2:** Powder properties.

Scott Density, g/cm^3^	Tap Density, g/cm^3^	Subsieve Auto Sizer		Laser Diffraction, µm					Hydrogen Loss, %
		Mean Particle Size, µm	Specific Surface Area, m^2^/g	D10	D50	D90	D99	D[4,3]	
1.61	2.78	1.85	0.41	0.64	2.24	6.47	12.13	2.95	0.99

**Table 3:** Mean values of green and as-sintered densities ^1^.

Compaction Pressure, MPa	Green Density, g/cm^3^	As-Sintered Density, g/cm^3^			
		850 °C	874 °C	898 °C	924 °C
200	4.50 (57.3%)	-	-	7.50 (95.4%)	-
400	5.14 (65.4%)	7.42 (94.4%)	7.56 (96.2%)	7.60 (96.7%)	7.62 (97.0%)
600	5.68 (72.3%)	-	-	7.71 (98.1%)	-

^1^ the values in brackets represent densities relative to TD = 7.86 g/cm^3^ (ThermoCalc).

**Table 4:** Bending properties of samples compacted at 400 MPa and sintered ^2^.

Sintering Temperature, °C	σ_BS,_ MPa	σ_0.2__,_ MPa	ε_pl_, %
850	997 ± 562	-	-
874	1290 ± 6	1284 ± 8	0.4 ± 0.2
898	1292 ± 8	1267 ± 77	1.3 ± 3.7
924	1328 ± 321	1239 ± 7	3.5 ± 8.4

^2^ confidence intervals were estimated at 90% confidence level throughout the article.

**Table 5:** Volume fractions of (Cu) and lattice parameters of ferrite in the powder and sintered samples.

Sintering Temperature, °C	V_(Cu),_ %	a_fe,_ Å
Powder	2.34	2.86582
850	1.12	2.86726
874	0.66	2.86699
898	0.76	2.86687
924	0.89	2.86737

**Table 6:** Effect of compaction pressure and sintering temperature on as-sintered hardness.

Compaction Pressure, MPa	HV1			
	850 °C	874 °C	898 °C	924 °C
200	-	-	234.8 ± 30.4	-
400	223.8 ± 20.7	246.5 ± 15.2	251.5 ± 13.4	233.0 ± 11.4
600	-	-	271.8 ± 8.9	-

**Table 7:** Effect of sintering temperature on porosity, pore size, and shape.

Sintering Temperature, °C	Porosity, Vol.%	Arithmetic Mean Pore Area, µm^2^	P_A_, µm^−2^	Pore Circularity	
				Arithmetic Mean	Weighted Mean ^3^
850	4.03 ± 0.94	0.30	0.13	0.562	0.484
874	3.12 ± 1.11	0.29	0.11	0.574	0.522
898	2.48 ± 0.57	0.27	0.09	0.597	0.566
924	3.10 ± 1.05	0.46	0.07	0.647	0.628

^3^ the fraction area of each pore has been taken as the weight.

**Table 8:** Chemical composition of sintered samples assessed by EDS.

Cu		Ni	P	Fe
**wt.%**	vol.%	wt.%	wt.%	bal.
3.37 ± 1.61	2.98 ± 1.43	1.18 ± 0.58	0.74 ± 0.10	bal.

## Data Availability

The data presented in this study are available on request from the corresponding author.
